# Single-port inflatable mediastinoscopic esophagectomy is a cure for esophageal cancer patients: Case report

**DOI:** 10.1097/MD.0000000000031619

**Published:** 2022-11-18

**Authors:** Xiayimaierdan Yibulayin, Keming Xu, Waresijiang Yibulayin, Abulimiti Abulaiti, Zhenhua Wu, Dan He, Anpeng Ran, Lei Ma, Xiaohong Sun

**Affiliations:** a Department of Thoracic Surgery, Affiliated Tumor Hospital of Xinjiang Medical University, Urumqi, China.

**Keywords:** cancer, esophageal squamous cell cancer, single-port inflatable mediastinoscopic esophagectomy

## Abstract

It is often difficult to perform transthoracic esophagectomy (TTE) in patients with chest deformities, as these patients may be lost to surgery for non-oncological reasons.

**Patient concerns:**

In this case, we had a patient with esophageal squamous cell carcinoma (ESCC) who was not suitable for TTE because of extensive thoracic adhesions caused by the left pneumonectomy 8 years ago.

**Diagnoses:**

ESCC.

**Interventions:**

Based on Professor Fujiwara’s surgical method, we further improved it by proposing a single-port inflatable mediastinoscopy combined with laparoscopic-assisted esophagectomy.

**Outcomes:**

At the time of this writing, computed tomography and gastroscopy revealed no stenosis of anastomosis, and no evidence of disease recurrence.

**Lessons:**

To the best of our knowledge, the present case is the first single-port inflatable mediastinoscopic esophagectomy performed on a patient undergoing pneumonectomy.

## 1. Introduction

Esophageal cancer is not uncommon worldwide, which has a high incidence and a poor prognosis.^[1]^ Despite the fact that in recent years the incidence and mortality rate of esophageal cancer has decreased, China still has one of the highest incidences of esophageal cancer in the world.^[2]^ Various methods have been brought in as the mainstream of treatment, including surgical and non-surgical palliative approaches, but surgical resection remains the preferred option, provided the patient is functionally operable.^[3]^ Open esophagectomy is a standard surgical procedure, but, because of its better prognosis and shorter hospital stays, nearly 45% of patients around the world are estimated to undergo the procedure using minimally invasive esophagectomy (MIE).^[4,5]^ However, pain and collapse of the lung due to thoracotomy may cause serious respiratory complications in elderly or comorbid patients. In addition, it is often difficult to perform (transthoracic esophagectomy [TTE]) in patients with cardiopulmonary dysfunction, previous history of surgery thoracic surgery and pleurisy, and chest deformity, etc, as these patients may be lost to surgery for non-oncological reasons.

Compared with TTE, non-TTE provides effective relief of postoperative pain, accelerates postoperative recovery, and reduces perioperative cardiopulmonary complications, thereby expanding the indications for surgery in patients who are not able to undergo TTE. There is single-port mediastinoscopic lymphadenectomy performed by Fujiwara in 2014 and further improved by Cao.^[6,7]^ This procedure allows efficient dissection of upper mediastinal lymph nodes and shows a favorable perioperative prognosis. This non-TTE procedure has the advantage of being less affected by thoracic conditions and therefore may be potentially beneficial for patients who fail to undergo conventional esophagectomy.

In this case, we had a patient with Esophageal Squamous Cell Carcinoma (ESCC) who was not suitable for TTE because of extensive and severe thoracic adhesions caused by the left pneumonectomy 8 years ago. Based on Professor Fujiwara’s surgical method, we further improved it by proposing a single-port inflatable mediastinoscopy combined with laparoscopic-assisted esophagectomy. The purpose of our application is to allow one to receive radical treatment.

## 2. Case presentation

**Figure 1.**
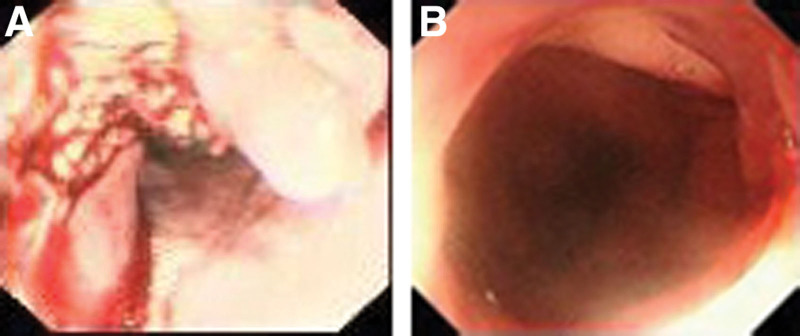
Preoperative and postoperative electronic gastroscopy of patient (A) Ulcer growth was visible in esophagus after the microscope into it 35 to 41 cm. (B) postoperative electronic gastroscopy revealed no development of anastomotic leakage and anastomosis.

**Figure 2.**
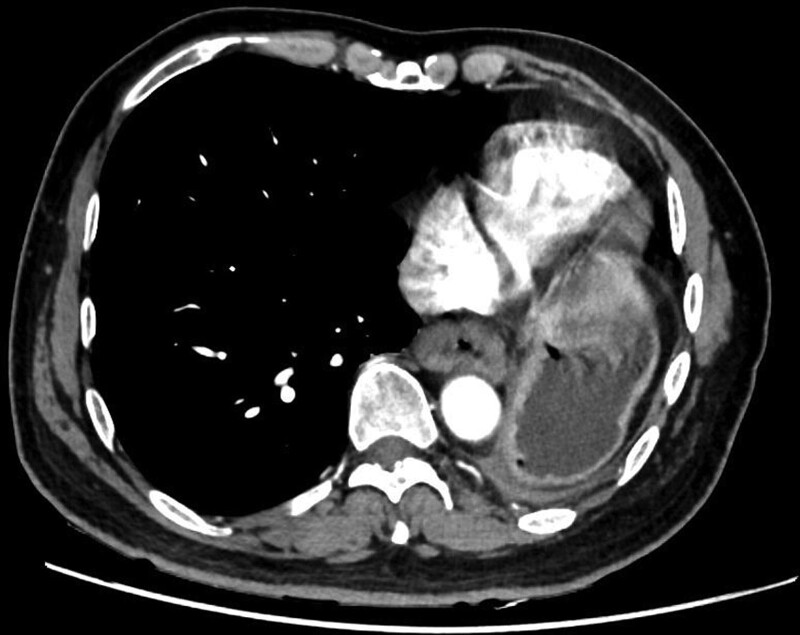
CT mediastinum window revealed a neoplasm, which was located in lower thoracic esophagus, measuring 5 × 3 cm in size as white arrow indicated. CT = computed tomography.

**Figure 3.**
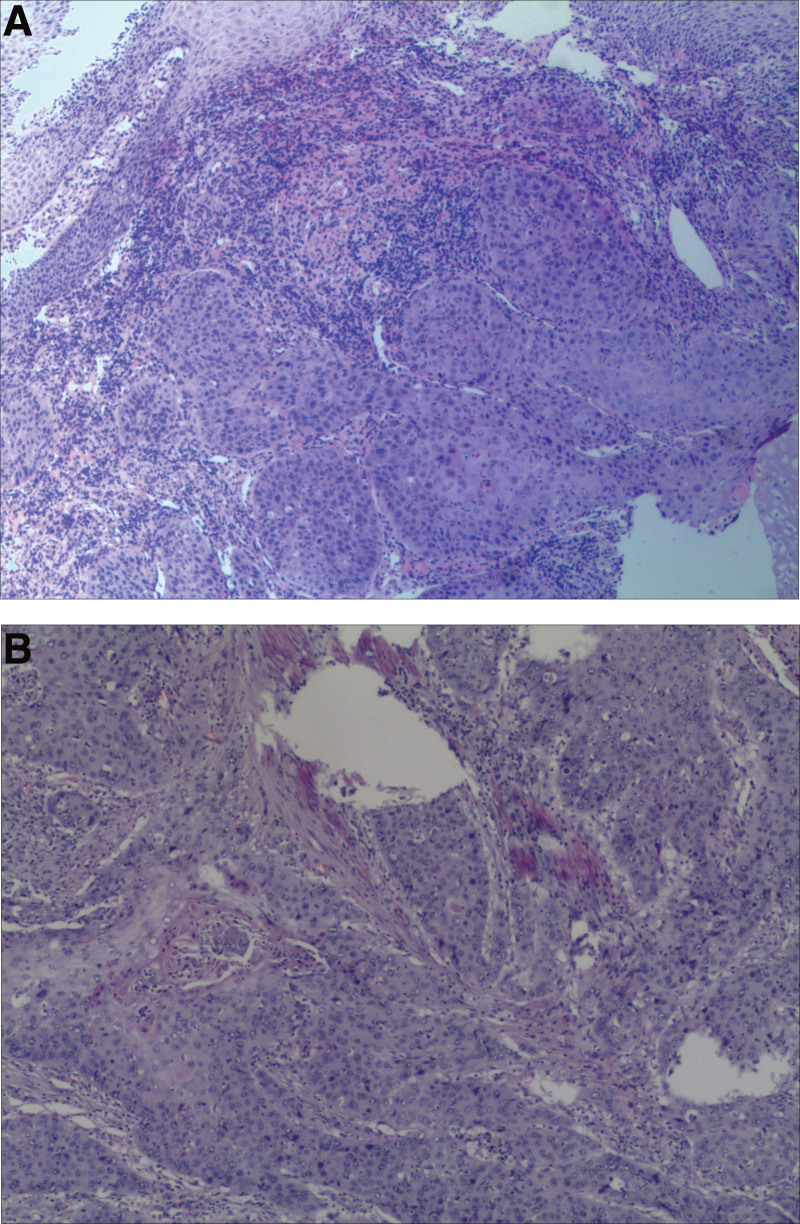
Preoperative (A) and postoperative (B) H&E staining of ESCC. (A) 40×. (B) 400×. ESCC = esophageal squamous cell carcinoma.

We report here the case of a 54-year-old male who presented with aggravated dysphagia and fatigue. There was no abnormality indicated in his family and social history. However, the left pneumonectomy he had undergone 8 years ago was of great significance. For this reason, to minimize the probability of intraoperative complications, he accepted a thorough evaluation prior to surgery. Ulcer growth visible in esophagus after the microscope into 35-41 cm (Fig. 1A), and computed tomography (CT) revealed a neoplasm, which was located in lower thoracic esophagus, measuring 6 cm in length (Fig. 2). Fine needle biopsy of the lesion revealed ESCC (Fig. 3A). Pulmonary function test revealed that FEV1 was 1.81, FEV1/FVC% 75, MVV73. Having ruled out surgical contraindications, the patient underwent single-port inflatable mediastinoscopy combined with laparoscopic-assisted esophagectomy, as described previously. The smooth and successful surgical procedure allowed the patient hospitalized for 20 days without complications and a postoperative staging of pT3N0M0 (Fig. 3B). The patient did not develop anastomotic leakage (Fig. 1B), pneumonia or other serious complications. At the 1-year follow-up, CT and gastroscopy revealed no stenosis of anastomosis, and no evidence of disease recurrence. At present, the patient is still being followed.

## 3. Discussion

Secondary surgery, following chest surgery, pleurisy, and thoracotomy for patients with chest deformities, has always posed a great challenge to thoracic surgeons. Radical surgical treatment of these patients is limited by the loss of normal anatomy, the difficulty in accessing the pleural cavity, and the inability to tolerate thoracotomy. By the time these patients have esophageal cancer, they are often deprived of the opportunity to have radical surgery.

Yet, non-TTE is an acceptable option. The conventional esophageal resection methods without opening the chest can reduce cardiopulmonary complications. However, the main metastasis pathway of esophageal cancers in the upper mediastinum is to spread to the lymph nodes along the recurrent laryngeal nerve. Dissection of lymph nodes along the bilateral recurrent laryngeal nerve is thus an indispensable part of radical esophagectomy. These approaches, mentioned above, unfortunately, are limited by the narrow surgical field of view and the poor results of upper mediastinal lymph node dissection. As a result, is used for intramediastinal lymph node sampling or biopsies for accurate staging diagnosis, rather than for total lymphadenectomy, which is essential for radical surgery for ESCC. In 2016, the first case of “Single-port inflatable mediastinoscopy combined with laparoscopy for the radical treatment of esophageal cancer” was completed by Professor Cao.^[7]^ This non-TTE may offer a potential for surgical treatment for patients who cannot undergo TTE, giving this group of esophageal cancer patients a chance for cure.

Based on our experiences, non-TTE, which greatly reduces the surgical trauma to patients, has great advantages over transthoracic surgery. The novel inflatable mediastinoscopy enables a clear surgical field, thus resulting in a great improvement in surgical safety; meanwhile, it also makes the total meso esophagus excision become possible, thus meeting the oncological requirements of the radical treatment of esophageal cancer; The surgery does not require to open the thoracic cavity, which significantly decreases the incidences of cardiopulmonary complications and renders the radical treatment less invasive; In comparison with radical esophageal cancer treatment by thoractic-abdominal endoscopic or conventional TTE, the clear surgical view is effective in shortening the operation time and reducing intraoperative bleeding.

Some shortcomings of this novel surgical method deserve to be mentioned. This surgical method was started in May 2018, hence, its long-term prognosis is still unknown. Secondly, the mediastinum is less extensive than the thorax. A successful surgery in the narrow surgical field is highly dependent on the experience of the surgeon. In the following study, a well-designed, randomized controlled trial are expected to be conducted to comprehensively evaluate the therapeutic efficacy and safety of this new surgical method. All these disadvantages are to be meticulously addressed in future studies.

## 4. Conclusion

Our results suggest that this surgery of single-port inflatable mediastinoscopy combined with laparoscopy radical esophageal resection for squamous cell carcinoma is safe and feasible. To the best of our knowledge, the present case is the first single-port inflatable mediastinoscopic esophagectomy performed on a patient undergoing pneumonectomy. It may offer a treatment idea for some patients who are unable to tolerate traditional esophagectomy, and a few patients may benefit from it.

## Author contributions

**Conceptualization:** Waresijiang Yibulayin.

**Data curation:** Xiayimaierdan Yibulayin.

**Methodology:** Keming Xu.

**Resources:** Abulimiti Abulaiti.

**Software:** Zhenhua Wu, Anpeng Ran.

**Visualization:** Dan He, Lei Ma.

**Writing – original draft:** Xiayimaierdan Yibulayin.

**Writing – review & editing:** Xiayimaierdan Yibulayin, Xiaohong Sun.
